# Anti-EGFR antibody sensitizes colorectal cancer stem-like cells to Fluorouracil-induced apoptosis by affecting autophagy

**DOI:** 10.18632/oncotarget.13233

**Published:** 2016-11-09

**Authors:** Ye Feng, Shuohui Gao, Yongjian Gao, Xuefeng Wang, Zhi Chen

**Affiliations:** ^1^ Department of Gastrointestinal Colorectal and Anal Surgery, China-Japan Union Hospital of Jilin University, Changchun 130033, China; ^2^ Department of Nephrology, First Hospital of Jilin University, Changchun 130021, China

**Keywords:** colorectal carcinoma (CRC), cancer stem cells (CSCs), CD133, CD44, EphB2

## Abstract

Recent reports suggest that colorectal carcinoma (CRC) may be sustained by a small subpopulation of cells, termed cancer stem cells (CSCs), which have drug resistance properties as a key reason for chemotherapy failure. The epidermal growth factor receptor (EGFR) controls CRC initiation and progression. Monoclonal antibody against EGFR (cetuximab) has been used in treatment of several cancers. However, the effects of cetuximab on CSCs in the CRC chemotherapy remain unclear. Here, we studied the effects of cetuximab on the CSC-like cells in Fluorouracil (5-FU)-treated CRC cells. CSC-like cells were independently isolated from CRC cells using CD133, CD44 or EphB2-high as markers and confirmed by tumor sphere formation assay. We found that 5-FU increased the apoptotic death of CSC-like CRC cells. Co-application of cetuximab augmented the apoptotic death of CSC-like CRC cells by 5-FU, seemingly through inhibition of 5-FU-induced increases in cell autophagy in CSC-like CRC cells. Together, our data suggest that EGFR monoclonal antibody may sensitize CSC-like CRC cells to 5-FU-induced apoptosis by affecting autophagy.

## INTRODUCTION

Colorectal carcinoma (CRC) is the third common cancer in humans [1–3]. Although the primary CRC are highly curable, some CRC may migrate to distal tissues, resulting in poor prognosis [4]. Recent reports suggest that CRC may be sustained by specific cells called cancer stem cells (CSCs). CRCs have potential innate drug resistance properties, leading to chemotherapy failure [4]. Moreover, CSCs are supposed to be responsible for the majority of the cancer invasiveness and metastases, which highlights the significance of treating CSCs rather than the complete cancer mass during cancer therapy [4].

CSCs are highly tumorigenic, and often play critical roles in cancer relapse and metastases [5–8]. Hence, treatments targeting CSCs may substantially improve the therapy [5–8]. Cell surface markers are pretty critical for isolating CSCs using a cell biology technology called flow cytometry. Importantly, although many cancers share same CSC markers, some CSC markers appear to be cancer-specific. In CRC, the best established ones are prominin-1 (CD133) [9–11], CD44 [12–14], and EphB2 [15–17]. However, the current identification of CRCs in CRC is not satisfactory [18–24].

Autophagy is a catabolic pathway for degradation and recycling of the cellular compartments for cell survival at harsh environments, using regularly by cancer cells to improve survival against chemotherapy [25–27]. During autophagy, a cytosolic form of microtubule-associated protein 1A/1B-light chain 3 (LC3-I) conjugates to form LC3-phosphatidylethanolamine conjugate (LC3-II), as a feature of autophagic activities [25–27]. Among all proteins that regulate autophagy, autophagy-associated protein 6 (ATG6, or Beclin-1) plays a pivotal role [28].

The epidermal growth factor receptor (EGFR) signaling pathway is involved in the initiation and progression of CRC [29–32]. Cetuximab is a FDA-approved EGFR chemeric human-murine monoclonal antibody against EGFR (cetuximab). However, the effects of cetuximab on CSCs in the chemotherapy of CRC remain unclear.

Here, we studied the effects of cetuximab on the CSC-like cells in Fluorouracil (5-FU)-treated CRC cells. CSC-like cells were independently isolated from CRC cells using CD133, CD44 or EphB2-high as markers and the features of these cells as CSC-like cells were proven by tumor sphere formation assay. We found that 5-FU increased the apoptotic death of CSC-like CRC cells, in an CCK-8 assay and an apoptotic assay. Co-application of cetuximab augmented the apoptotic death of CSC-like CRC cells by 5-FU, seemingly through inhibition of 5-FU-induced increases in cell autophagy in CSC-like CRC cells. Together, our data suggest that EGFR monoclonal antibody may sensitize CSC-like CRC cells to 5-FU-induced apoptosis by affecting autophagy.

## RESULTS

### CD133-positive cells are enriched with CSCs in CRC

In order to examine the effects of cetuximab on the CSC population of CRC cells treated with chemotherapeutic drugs (e.g. 5-FU), we isolated CSC-like cells from CRC cell lines using different CSC markers, CD133, CD44 and EphB2, independently. We chose two CRC cell lines, HT-29 and SW480, in our study. HT-29 cells express low levels of EGFR, and has wild-type KRAS. On the other hand, SW480 express high levels of EGFR, and has mutated KRAS [37]. Hence, these two lines are very good representatives in analyzing the effects of cetuximab on the CSC population of CRC cells treated with 5-FU.

**Figure 1:**
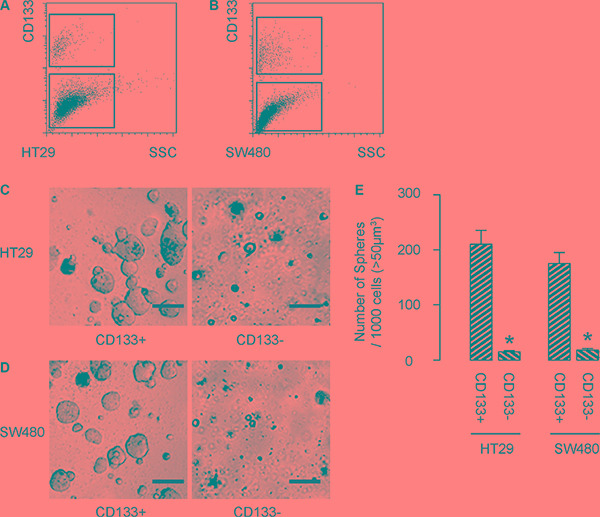
CD133-positive cells are enriched with CSCs in CRC (**A**–**B**) We isolated CD133+ cells vs CD133− cells from either HT-29 cells (A), or SW480 cells (B). (**C**–**E**) To confirm that CD133+ cells may be enriched for CSCs, we performed tumor sphere formation assay, shown by representative images in either HT-29 cells (C), or SW480 cells (D), and by quantification (E). *p < 0.05. *N* = 5. Scale bars are 50 μm.

First, we isolated CD133+ cells vs CD133− cells from either HT-29 cells (Figure 1A), or SW480 cells (Figure 1B). To confirm that CD133+ cells may be enriched for CSCs, we performed tumor sphere formation assay. We found that CD133+ cells formed significantly more spheres than CD133− cells, in either HT-29 cells (Figure 1C), or SW480 cells (Figure 1D). Quantification was shown in Figure 1E. Hence, CD133-positive cells are enriched with CSCs in CRC.

### CD44-positive cells are enriched with CSCs in CRC

**Figure 2:**
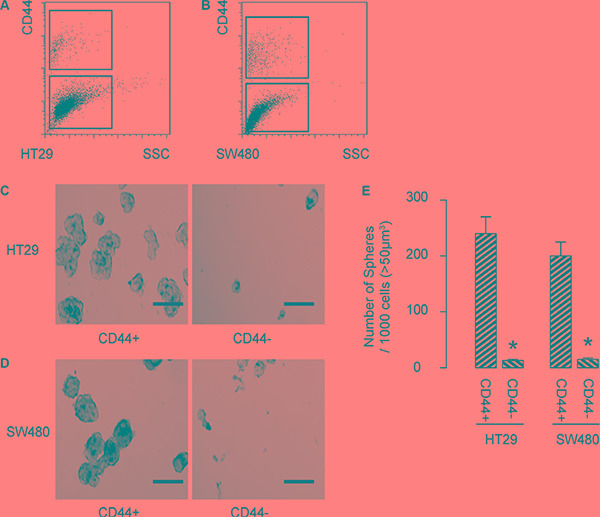
CCD44-positive cells are enriched with CSCs in CRC (**A**–**B**) We isolated CD44+ cells vs CD44− cells from either HT-29 cells (A), or SW480 cells (B). (**C**) To confirm that CD44+ cells may be enriched for CSCs, we performed tumor sphere formation assay, shown by representative images in either HT-29 cells (C), or SW480 cells (**D**) and by quantification (**E**). *p < 0.05. *N* = 5. Scale bars are 50 μm.

Then, we isolated CD44+ cells vs CD44− cells from either HT-29 cells (Figure 2A), or SW480 cells (Figure 2B). To confirm that CD44+ cells may be enriched for CSCs, we performed tumor sphere formation assay. We found that CD44+ cells formed significantly more spheres than CD44− cells, in either HT-29 cells (Figure 2C), or SW480 cells (Figure 2D). Quantification was shown in Figure 2E. Hence, CD44-positive cells are enriched with CSCs in CRC.

### EphB2-high cells are enriched with CSCs in CRC

**Figure 3:**
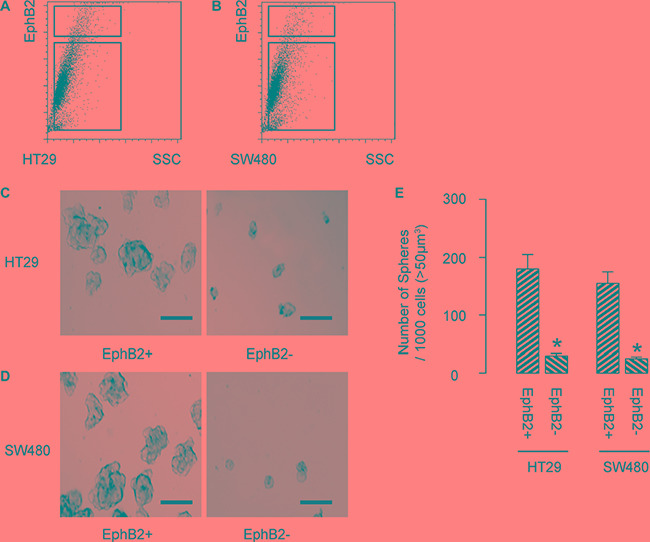
EphB2-high cells are enriched with CSCs in CRC (**A**–**B**) We isolated EphB2-high cells vs EphB2-low cells from either HT-29 cells (A), or SW480 cells (B). (**C**) To confirm that EphB2-high cells may be enriched for CSCs, we performed tumor sphere formation assay, shown by representative images in either HT-29 cells (C), or SW480 cells (**D**) and by quantification (**E**). *p < 0.05. *N* = 5. Scale bars are 50 μm.

Finally, we isolated EphB2-high cells vs EphB2-low cells from either HT-29 cells (Figure 3A), or SW480 cells (Figure 3B). To confirm that EphB2-high cells may be enriched for CSCs, we performed tumor sphere formation assay. We found that EphB2-high cells formed significantly more spheres than EphB2-low cells, in either HT-29 cells (Figure 3C), or SW480 cells (Figure 3D). Quantification was shown in Figure 3E. Hence, EphB2-high cells are enriched with CSCs in CRC. Thus, these enriched CSC-populations (CD133+; CD44+; EphB2-high) were independently used for analyzing the effects of cetuximab on the CSC population of CRC cells treated with 5-FU.

### EGFR inhibition increases 5-FU-induced apoptotic death in CSC-like CRC cells

**Figure 4:**
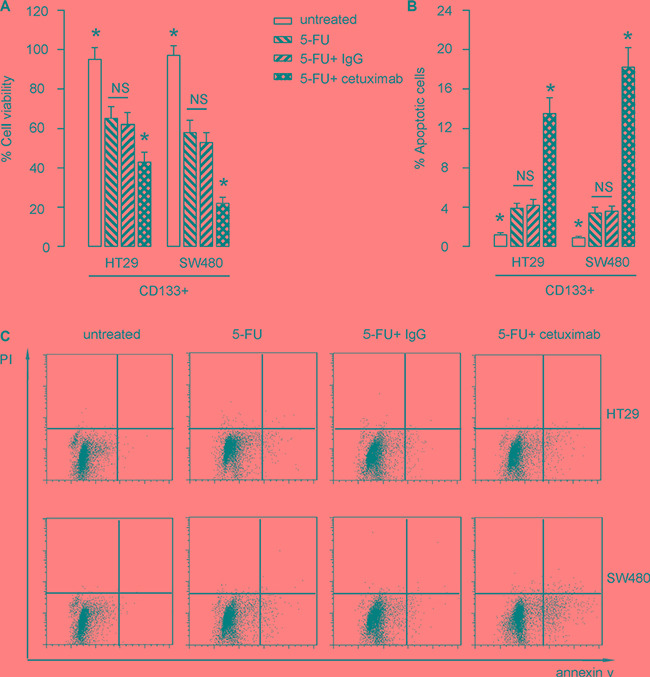
EGFR inhibition increases 5-FU-induced apoptotic death in CSC-like CRC cells (**A**–**C**) Cultured CD133+ HT-29 cells, or SW480 cells were treated with/without 5-FU. Moreover, the 5-FU-treated cells were also treated with cetuximab, or control IgG. After 24 hours, the cells were analyzed. (A) CCK-8 assay. (B–C) The apoptosis assay, shown by representative flow charts (B), and by quantification (C). *p < 0.05. NS: non-significant. *N* = 5.

Cultured CD133+ HT-29 cells, or SW480 cells were treated with/without 5-FU. Moreover, the 5-FU-treated cells were also treated with cetuximab, or control IgG. After 24 hours, the cells were analyzed. We found that 5-FU significantly reduced the cell viability of CD133+ CRC cells, in an CCK-8 assay (Figure 4A), seemingly by increasing the apoptotic cell death (Figure 4B–4C). Co-application of cetuximab augmented the apoptotic death of CD133+ CRC cells by 5-FU (Figure 1A–1C). We got similar results, using CD44+ or EphB2 cells in this study (not shown). Thus, EGFR inhibition increases 5-FU-induced apoptotic death in CSC-like CRC cells.

### EGFR inhibition reduces 5-FU-induced cell autophagy in CSC-like CRC cells

**Figure 5:**
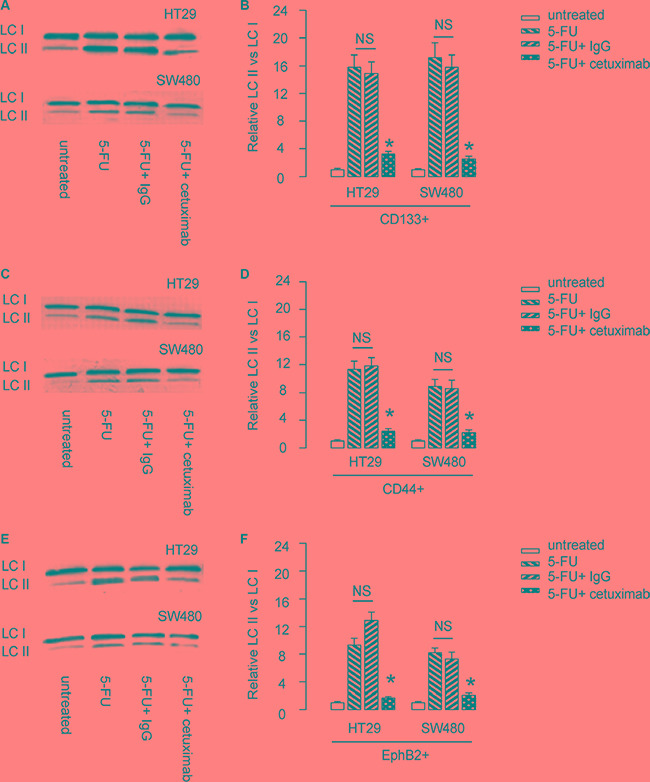
EGFR inhibition reduces 5-FU-induced cell autophagy in CSC-like CRC cells (**A**–**B**) LCII vs LC I levels of CD133+ CRC cells were analyzed by Western blot, shown by representative blots (A), and by quantification (B). (**C**–**D**) LCII vs LC I levels of CD44+ CRC cells were analyzed by Western blot, shown by representative blots (C), and by quantification (D). (**E**–**F**) LCII vs LC I levels of EphB2-high CRC cells were analyzed by Western blot, shown by representative blots (E), and by quantification (F). *p < 0.05. NS: non-significant. *N* = 5.

**Figure 6:**
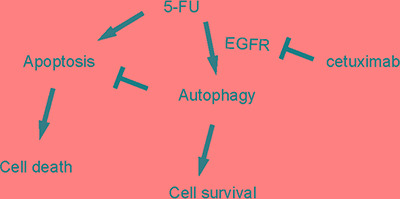
Schematic of the model 5-FU may not only induce apoptotic cell death of CSC-likes in CRC, but also induce cell autophagy to contradict apoptotic cell death to allow some cells to survive the treatment. However, cetuximab may inhibit the autophagy to increase the sensitivity of CSC-like CRC cells to chemotherapy.

Since autophagy and apoptosis are closely related and may affect each other at molecular level, we thus examined whether EGFR inhibition may alter cell autophagy in 5-FU-treated CSC-like CRC cells. LCII vs LC I levels are a golden standard for evaluating cellular autophagy activity. We found that 5-FU induced cell autophagy in CD133+ CRC cells, which was significantly attenuated by cetuximab (Figure 5A–5B). Similar results were obtained when we used either CD44+ cells, or EphB2-high cells (Figure 5C–5F). Thus, 5-FU may not only induce apoptotic cell death of CSC-likes in CRC, but also induce cell autophagy to contradict apoptotic cell death to allow some cells to survive the treatment. However, cetuximab may inhibit the autophagy to increase the sensitivity of CSC-like CRC cells to chemotherapy (Figure 6).

## DISCUSSION

CSCs are characterized by their drug resistance properties. Treatments targeting CSCs thus could improve the therapeutic outcome of rapidly growing cancers and highly metastatic cancers [5–8].

In the current study, we showed that 5-FU treated CRC cells underwent apoptotic cell death. However, the effects of 5-FU on CRC cells could be augmented by co-treatment of the CRC cells with cetuximab, which has been widely used in treating malignant cancer in clinic. Here, we examined two CRC cell lines, among which SW480 has mutated KRAS while HT29 has wild-type KRAS. Those cell lines thus represent CRC cells with or without mutated KRAS. Since mutated KRAS is resistant to EGFR-targeted mAbs [38], our data that show co-application of cetuximab with 5-FU is effective for both lines may suggest that the effects of cetuximab on cell death are indirectly and may result from the changes of sensitivity of CRC cells to 5-FU. This conclusion is also supported by the data that a control group of cetuximab alone of same dosage did not have significant effects on cell death.

Interestingly, we found that the effects of cetuximab on CRC cells appeared to be on CSC-like cells, which were independently isolated from CRC cells using CD133, CD44 or EphB2-high as markers. Of note, these markers have been used to enrich CSC cells, but not purify them. Using these markers independently increased the reliability of the conclusion on CSC cells, which were further proved by tumor sphere formation assay, which is a gold method for validating CSCs. We found that 5-FU increased the apoptotic death of CSC-like CRC cells in an apoptotic assay. Moreover, the changes in cell number were confirmed in a CCK-8 assay.

Then, we studied the mechanisms underlying the cetuximab augmented CRC cell death. We found that 5-FU treatment decreased CRC cell viability in a dose-dependent manner. However, this 5-FU-induced CRC cell death appeared to be attenuated by augmentation in autophagy-associated cell survival. Thus, 5-FU may induce both apoptotic cell death and autophagic cell survival in CRC cells, while autophagy could be a negative feedback from CRC cells to resist 5-FU. In another word, 5-FU damaged CRC cells, the CSC-like cells from which upregulated autophagy associated proteins to enhance autophagic cell survival against the effects of 5-FU. The cetuximab treatment inhibited the autophagy of CSC-like cells from CRC, resulting in improved elimination of the CRC. A limitation of the current study is that the effect of cetuximab/5-FU adjunctive treatment *in vivo* was not investigated, which should be addressed in future studies. Moreover, further dissection of the details of the involved signaling pathway is highly needed.

Together, our data suggest that EGFR monoclonal antibody may sensitize CSC-like CRC cells to 5-FU-induced apoptosis by affecting autophagy. Cetuximab treatment may be a promising treatment targeting chemo-resistance of CSC-likes from CRC.

## MATERIALS AND METHODS

### Protocol approval

All the experimental methods in the current study has been approved by the research committee at Jilin University. All the experiments have been carried out in accordance with the guidelines from the research committee at Jilin University.

### Cell line culture and treatment

From all published CRC cell lines, we selected HT-29 and SW480 in our study. HT-29 is a colorectal adenocarcinoma from a 44 year-old female, and has been describe before [33]. SW480 is a colorectal adenocarcinoma from a 50 year-old male, and has been describe before [34]. Both lines were purchased from American Type Culture Collection (ATCC, Rockville, MD, USA), and maintained in Dulbecco's modified Eagle's medium (DMEM, Invitrogen, Carlsbad, CA, USA) supplemented with 15% fetal bovine serum (FBS; Sigma-Aldrich, St Louis, MO, USA) in a humidified chamber with 5% CO_2_ at 37°C. 5-FU (Sigma-Aldrich) was prepared in a stock of 1 mmol/l and applied to the cultured CRC cells at 2 μmol/l [35, 36]. Cetuximab is an EGFR chemeric human-murine monoclonal antibody, and was applied to the cultured CRC cells at 0.5 mg/ml. Isotype-match IgG of same concentration was used as a control.

### Primary tumor sphere culture

Purified CSC-like cells by flow cytometry were washed, acutely dissociated in oxygenated artificial cerebrospinal fluid and subject to enzymatic dissociation. Cells were then re-suspended in tumor sphere media (TSM) consisting of a serum-free DMEM, human recombinant EGF (20 ng/ml; Sigma-Aldrich), bFGF (20 ng/ml; Sigma-Aldrich), leukemia inhibitory factor (10 ng/ml; Sigma-Aldrich) and N-acetylcysteine (60 μg/ml; Sigma-Aldrich), and then plated at a density of 2 × 10^6^ cells/60 mm plate.

### Analysis of CD133, CD44, and EphB2 by flow cytometry

CD133, CD44, and EphB2 -based cell analysis and sorting were performed by flow cytometry, using either PEcy7-conjugated anti-human CD133 antibody, FITC-conjugated anti-human CD44 antibody or APC-conjugated anti-human EphB2 antibody (Becton-Dickinson Biosciences, San Jose, CA, USA), respectively. Flow cytometry was performed using a FACSAria (Becton-Dickinson Biosciences) flow cytometer, and analyzed with Flowjo software (Flowjo LLC, Ashland, OR, USA).

### Cell viability assay

The CCK-8 detection kit (Sigma-Aldrich) was used to measure cell viability according to the manufacturer's instructions. Briefly, cells were seeded in a 96-well microplate at a density of 5 × 10^4^/ml. After 24 h, cells were treated with resveratrol. Subsequently, CCK-8 solution (20 ml/well) was added and the plate was incubated at 37°C for 2 h. The viable cells were counted by absorbance measurements with a monochromator microplate reader at a wavelength of 450 nm. The optical density value was reported as the percentage of cell viability in relation to the control group (set as 100%).

### Apoptosis assay

Cells were labeled with annexin V-FITC and propidium iodide (PI), and then examined with an apoptosis detecting kit (Invitrogen) for apoptosis. Samples were analyzed by flow cytometry and the results were analyzed by CellQuest software (Becton-Dickinson Biosciences).

### Western blot

Protein was extracted from the cultured cells with RIPA lysis buffer (1% NP40, 0.1% Sodium dodecyl sulfate (SDS), 100 μg/ml phenylmethylsulfonyl fluoride, 0.5% sodium deoxycholate, in PBS) on ice. The supernatants were collected after centrifugation at 12000× g at 4°C for 20 min. Protein concentration was determined using a BCA protein assay kit (Bio-rad, China), and whole lysates were mixed with 4 × SDS loading buffer (125 mmol/l Tris-HCl, 4% SDS, 20% glycerol, 100mmol/l Dithiothreitol (DTT), and 0.2% bromophenol blue) at a ratio of 1:3. Samples were heated at 100°C for 5 min and were separated on SDS-polyacrylamide gels. The separated proteins were then transferred to a PVDF membrane. The membrane blots were first probed with a primary antibody. After incubation with horseradish peroxidase-conjugated second antibody, autoradiograms were prepared using the enhanced chemiluminescent system to visualize the protein antigen. The signals were recorded using X-ray film. Primary antibodies were rabbit anti-LC (Cell Signaling, San Jose, CA, USA). Secondary antibody is HRP-conjugated anti-rabbit (Jackson ImmunoResearch Labs, West Grove, PA, USA). LC I was used as an internal control for LC II.

### Statistical analysis

All statistical analyses were carried out using the SPSS 18.0 statistical software package. All data were statistically analyzed using one-way ANOVA with a Bonferroni correction, followed by Fisher's exact test to compare two groups. All values in cell and animal studies are depicted as mean ± standard deviation and are considered significant if *p* < 0.05.
